# Recent Advances on Multi-Parameter Flow Cytometry to Characterize Antimicrobial Treatments

**DOI:** 10.3389/fmicb.2016.01225

**Published:** 2016-08-08

**Authors:** Lucie Léonard, Lynda Bouarab Chibane, Balkis Ouled Bouhedda, Pascal Degraeve, Nadia Oulahal

**Affiliations:** Univ Lyon, Université Claude Bernard Lyon 1, ISARA Lyon, BioDyMIA (Bioingénierie et Dynamique Microbienne aux Interfaces Alimentaires), Equipe Mixte d'Accueil n°3733, IUT Lyon 1Bourg en Bresse, France

**Keywords:** multi-parameter flow cytometry, microorganisms, antimicrobial treatment, double-staining, antimicrobial mechanism, viability, culturability

## Abstract

The investigation on antimicrobial mechanisms is a challenging and crucial issue in the fields of food or clinical microbiology, as it constitutes a prerequisite to the development of new antimicrobial processes or compounds, as well as to anticipate phenomenon of microbial resistance. Nowadays it is accepted that a cells population exposed to a stress can cause the appearance of different cell populations and in particular sub-lethally compromised cells which could be defined as viable but non-culturable (VBNC). Recent advances on flow cytometry (FCM) and especially on multi-parameter flow cytometry (MP-FCM) provide the opportunity to obtain high-speed information at real time on damage at single-cell level. This review gathers MP-FCM methodologies based on individual and simultaneous staining of microbial cells employed to investigate their physiological state following different physical and chemical antimicrobial treatments. Special attention will be paid to recent studies exploiting the possibility to corroborate MP-FCM results with additional techniques (plate counting, microscopy, spectroscopy, molecular biology techniques, membrane modeling) in order to elucidate the antimicrobial mechanism of action of a given antimicrobial treatment or compound. The combination of MP-FCM methodologies with these additional methods is namely a promising and increasingly used approach to give further insight in differences in microbial sub-population evolutions in response to antimicrobial treatments.

## Introduction

Inactivation of microorganisms by physical treatments [heat, Ultra-Violet (UV) light irradiation, supercritical carbon dioxide, high hydrostatic pressure,…] or by the action of antimicrobial compounds (biocides, organic acids, peptides, essential oils,…) can result from several mechanisms: inhibition of cell wall synthesis, disruption of the cytoplasmic membrane, binding to DNA, inhibition of protein synthesis, or anti-metabolite activity (Lee et al., 2015). To develop new antimicrobial processes or compounds in food or medical microbiology, an understanding of their mechanisms of action is vital in order to apply them best and to anticipate microbial resistance phenomenon.

Over the years, several methods have been developed to measure viability and vitality of microbes under various stresses: plating, slide culture, vital stains, metabolic activity monitoring, cell components monitoring, fermentation capacity, acidification potential, or oxygen uptake ability (Hayouni et al., 2008). Such methods are time-consuming and labor intensive (Wilkinson, 2015). Besides, when classical plate count method is used to assess survival of a bacterial population after exposure to an environmental stress, the viability is determined by counting live cells which are those that managed to replicate under the particular experimental conditions, while all the others will be presumed dead (Hayouni et al., 2008). Nowadays, it is well-documented that, under stress conditions a population will exhibit cell subpopulations with phenotypes that most likely escape this logic. Environmental stresses can trigger the occurrence of certain cell populations, called viable but non-culturable cells (VBNC), which were stressed and lost their ability to grow on agar medium, but still showed metabolic activity (Ananta et al., 2004). Quantification of injured cells is a great concern for microbiologists, as this subpopulation might be critical if cells can recover and revert to their physiologically active condition (Ayari et al., 2013). For these reasons, fluorescence techniques combined with direct optical detection methods for the rapid assessment of bacterial viability have been increasingly favored for about 10 years.

Among these techniques, multi-parameter flow cytometry (MP-FCM) has been shown to be a powerful tool for rapidly analyzing populations on a cell-by-cell basis and can be applied in many areas of food or medical microbiology (detection of pathogenic bacteria, monitoring lactic acid bacteria fermentation, rapid microbiological analysis of drinking water; Schenk et al., 2011). A flow cytometer can be described as an “automatic microscope” with the advantages of objectivity, high analysis rate, precision and sensitivity (Díaz et al., 2010). The principle is that particles in suspension are pumped into a narrow flow stream intersected by one or more laser beams. Single particles, such as microbial cells, are illuminated individually with the resulting light scatter and fluorescence emission detected at appropriate wavelengths (Bridier et al., 2015). A very large number of particles can be measured, 5000 cells per second in common and even up to 100,000 in specialized instruments, measuring multiple cellular parameters on each cell simultaneously (Díaz et al., 2010). Each individual cell can be characterized based on its fluorescence color, the intensity of the fluorescence signal, as well as the size, shape and granularity of the particles (Bridier et al., 2015). This method is highly compatible with a broad range of fluorescent stains and cell labeling methods. Díaz et al. (2010) detailed principles and instrumentation through schematic descriptions in their review about application of flow cytometry (FCM) to monitor industrial microbial bioprocess.

Firstly FCM developments occurred in human clinical applications especially for immunological analysis (Wilkinson, 2015). For less than 20 years, FCM has become an indispensable tool to the complex area of microbiology. In 2000, a set of publications in the Journal of Microbiological Methods presented cytometry for bacteria (Nebe-von-Caron et al., 2000; Shapiro, 2000; Steen, 2000). More recently, these first data were supplemented by a review of Tracy et al. (2010). The ability to use FCM to visualize, enumerate and analyze a population of cells into subpopulations of varying physiological status is a valuable aid to understanding this intricate area for the microbiologists. Bridier et al. (2015) reviewed the applications in food microbiology such as study of food bacteria function, detection of food microbial communities or detection and persistence of food pathogens. Moreover FCM can be used to elucidate antimicrobial mechanism in food or health domain. Recently, Mathur et al. (2016) published a review about FCM as a tool to study the effects of bacteriocins on prokaryotic and eukaryotic cells. The advancement of FCM and the introduction of novel fluorochromes allow to study the viability of cells, the membrane structure and its integrity, and the membrane potential at a single-cell level. In the perspective of elucidation of the antimicrobial mechanism, FCM should be a very interesting tool. In this review, we describe some recent studies that use FCM as a tool to evaluate the effect of antimicrobial treatment on microbial cells. More specifically, we focus on the use of MP-FCM with individual and simultaneous staining describing the advantages and the limitations. FCM is by definition a multi-parametric technique: cells are gated on at least size or complexity parameters and one parameter of fluorescence. Nevertheless, MP-FCM was defined here, as described in the literature, as FCM using several fluorochromes in the same study (Tracy et al., 2010). Based on recently published results, the complementarity with other methods and particularly plate counts methods is discussed.

## Informations resulting from direct analysis of microbial cells by flow cytometry

Although staining of microbial cells with dyes prior to flow cytometry analysis is dominating (and will be presented thereafter), direct analysis of cells without staining cells already gives information regarding the morphology of analyzed cells.

Even without staining the sample, a cell immersed in the injected solution already produces signals through the orthogonal-to-flow laser focused beam. The fraction of light scattered collected in the same direction as the incident light is known as Forward Angle Light Scatter (FALS) or Forward Scatter (FS or FSC). This fraction allows an estimation of the cell size: indeed, the quantity of the scattered light increases with the cell size (Díaz et al., 2010; Tracy et al., 2010). The fraction of light scattered laterally and fluorescence are collected and divided by a lens at 90° from the incidence axis of the laser. The fraction of light scattered in right angle is known as Right Angle Light Scatter (RALS) or Side Scatter (SS or SSC). This signal is related to cell complexity described by morphological characteristics such as cell surface roughness, cell membrane, nucleus and internal granular material, number of organelles (Díaz et al., 2010). For example, this first information concerning SSC vs. FSC allowed Kramer and Thielmann (2016) detecting that bacterial cells aggregated during heat exposure by change of the scatter signals. The strong increase of SSC and FSC indicated that cells formed large agglomerates, exhibiting up to 100-times higher scatter signals than single cells. Booyens and Thantsha (2014) also detected differences in shape and density of the populations' scatter patterns after exposure to garlic clove extract compared to control population for all the tested *Bifidobacterium* strains. Their hypothesis was that this change in size and external morphology was a change from rod to coccoid shape. Schenk et al. (2011) highlighted the same modification for *Escherichia coli* cells after UV-C light treatment.

## Analysis of microbial cells by flow cytometry after their staining with dyes

Additional information can be obtained provided that samples are stained using fluorochromes. Scattering and fluorescence signals provide information about intrinsic and extrinsic cell parameters, respectively.

### Multi-parameter flow cytometric analysis: individual use of dyes

A way to use the flow cytometry to characterize antimicrobial treatments is to perform a multi-parameter analysis with different stains in combination. This approach will be presented thereafter when these stains are used simultaneously (in 3.2. part). However, several researchers have chosen to use dyes separately (Table 1).

**Table 1:** **Examples of individual uses of dyes to perform a multi-parameter flow cytometry analysis in order to characterize antimicrobial mechanisms**.

**Reference**	**Objective**	**Dyes and their function**	**Main result**
Antolinos et al., 2014	Effect of acid shock on cell viability of *Bacillus cereus* and *Bacillus weihenstephanensis* vegetative cells	• PI (membrane permeability) • cFDA (esterase activity)	pH 3.4, 3.8, and 4.2 caused membrane disruption and subsequent bacterial cell death in the first 24 h exposition to these acidic environments.
Boda et al., 2015	Investigate the generated reactive oxygen species (ROS) causing peroxidation of the membrane lipids and ion channel proteins, leading to greater permeabilization of the bacterial membranes	• DCFH-DA [intracellular indicator of reactive oxygen species (ROS)]	~60 and ~70% reduction was recorded in the survival of staphylococcal species and *Escherichia coli*, respectively at pulsed magnetic field as evaluated by colony forming unit (CFU) analysis and flow cytometry. A 2–5 fold increase in intracellular ROS (reactive oxygen species) levels suggests oxidative stress as the key mediator in PMF induced bacterial death/injury.
Caldeira et al., 2013	Assessment of antibacterial properties of L-cysteine and mechanism of action against *Staphylococcus aureus* and *Klebsiella pneumoniae*	• PI (membrane permeability) • DiBAC_4_ (membrane potential) • CTC (respiratory activity)	The main mechanism of action of L-Cys on both bacteria, *K. pneumonia*, and *S. aureus*, was the reduction in metabolic activity, consistent with the results that confirmed the bacteriostatic effect of L-Cys on both these bacteria.
Kramer and Muranyi, 2014	Investigate the effects of a pulsed light treatment on the physiological properties of *Listeria innocua* and *Escherichia coli*	• PI (membrane permeability) • DiBAC_4_ (membrane potential) • EB (efflux pump activity) • cFDA (esterase activity)	Oxidative stress with concomitant damage to the DNA molecule was shown to be directly responsible for the loss of cultivability due to pulsed light rather than a direct rupture of cell membranes or inactivation of intracellular enzymes.
Kramer and Thielmann, 2016	Monitoring the live to dead transition of bacteria during thermal stress	• PI (membrane permeability) • DiBAC_4_ (membrane potential) • cFDA (esterase activity) • 2-NBGD (glucose uptake)	Exposure to moderate heat first of all compromised the function of the respiration chain and other heat sensitive proteins of the cell membrane such as efflux pumps. Membrane rupture and intracellular esterase activity were less affected and strong differences depending on the type of bacteria regarding their Gram-staining behavior were observed.
Lee et al., 2015	Mechanism of action of scopolendin 2 against *E. coli* O157 and *Candida albicans*	• Sytox Green • (membrane permeability) • DiBAC_4_ (membrane potential) • DiSC_3_ (membrane potential)	Scopolendin 2 led to the formation of pores in microbial plasma membrane, subsequent leakage of cytoplasmic matrix components and consequent membrane depolarization, ultimately resulting in microbial cell death.
Morishige et al., 2015	Analysis of the metabolic response of H_2_O_2_-treated *Salmonella* cells	• CTC (respiratory activity) • 2-NBGD (glucose uptake) • EdU-Alexa 488 • (DNA synthesis)	H_2_O_2_-treated *Salmonella* cells did not lose their biological activities of living cells all together. Different subpopulations of Viable But Non-Culturable bacteria were detected. Cells lost their DNA-synthesis activity first, then CTC-reducing activity, and finally glucose-uptake activity to a lesser extent.
Silva et al., 2011	*Coriandrum sativum* essential oil mode of action against *Candida* species	• PI (membrane permeability) • DiBAC_4_ (membrane potential) • DRAQ5 (DNA staining)	Coriander essential oil kills *Candida* spp. by damaging the cytoplasmic membrane, leading to an impairment of cellular functions.
Teng et al., 2014	Elucidate further the antimicrobial mechanism of AvBD103b, an avian defensin, on the *Salmonella enteritidis* CVCC3377 cell membrane and intracellular DNA	• PI (membrane permeability) • FITC-labeled AvBD103b • (AvBD103b permeation of the membrane)	Antimicrobial target of AvBD103b was the cell membrane.

*PI, Propidium Iodide; cFDA, carboxyFluorescein DiAcetate; DiBAC_4_, Bis-(1,3-DibutylBarbituric ACid) Trimethine Oxonol = bis-oxonol = BOX; CTC, 5-Cyano-2,3-ditolyl Tetrazolium Chloride; EB, Ethidium Bromide; 2-NBGD, 2-[N-(7-nitrobenz-2-oxa-1,3-Díazol-4-yl) amino]-2-deoxy-Dglucose; EdU: 5-ethynyl-2-deoxyuridine; FITC, fluorescein isothiocyanate; DRAQ5, 1,5-bis{[2-(di-methylamino) ethyl]amino}-4, 8-dihydroxyanthracene-9,10-dione; DiSC_3_, 3,3′-Dipropylthiadicarbocyanine Iodide, DCFH-DA, 2′,7′-dichlorofluorescein-diacetate*.

The following paragraphs briefly discuss microbial FCM dyes commonly employed in recent works to characterize antimicrobial treatments.

#### Membrane integrity

To interrogate membrane integrity, the nucleic acids (NA) content of individual cells is analyzed and dye exclusion methods are favored. NA dyes can stain DNA, RNA, or both (Tracy et al., 2010). Cells showing intact membranes are impermeable to multiple charges dyes such as dyes of the Sytox™ family or to cyanines such as TO-PRO®3. If cells lose membrane integrity, these dyes enter into the cells emitting fluorescence upon NA binding. Propidium iodide (PI) is the most commonly used dye (Díaz et al., 2010). This dye is usually employed for dead cells detection and it is suitable for multi-parametric analysis along with green fluorochromes such as SYTO9®. It contains two positive charges and is normally excluded from cells due to its divalence (Kim et al., 2009). Therefore, PI can only enter permeabilized cytoplasmic membranes. For instance, the commercial available LIVE/DEAD® BacLight™ kit from Molecular Probes is the most used (Possemiers et al., 2005; Kim et al., 2009; Muñoz et al., 2009; Martínez-Abad et al., 2012; Choi et al., 2013; Booyens and Thantsha, 2014; Fernandes et al., 2014; Manoil et al., 2014; Pal and Srivastava, 2014; Boda et al., 2015; Freire et al., 2015; Li H. et al., 2015; Li W. et al., 2015).

#### Pump activity

Ethidium bromide (EB) is a positively-charged monovalent compound that is used to evaluate the efflux pump system of bacteria. It is a membrane-permeant and it enters into intact cell membranes, but it is actively pumped out of the cell via a non-specific proton anti-port transport system (Kim et al., 2009; Díaz et al., 2010). When the membrane is damaged and the efflux pump also malfunctions, EB can stain the intracellular DNA of the cell (Kim et al., 2009).

#### Membrane potential

Membrane potential is generated due to the different ions content inside and outside the cell and it varies from 100 to 200 mV. Only living cells are able to maintain membrane potential: therefore, it is one of the most used parameters to assess cell viability (Díaz et al., 2010). When this difference decreases to zero, the membrane is structurally damaged, and ions go across the membrane freely, but if it means a decrease in cell activity, it does not necessarily imply death. Measurements are carried out by using lipophilic dyes which go through the cell membrane and accumulate according to their charge. The fluorescence signal can be directly related to cell energy levels and to test the reliability of staining, it is recommended to observe if the dye uptake is sensitive to ionophores such as carbonyl cyanide m-chlorophenylhydrazone (CCCP; Pianetti et al., 2008; Díaz et al., 2010; Hammer and Heel, 2012; Li W. et al., 2015).

Cationic dyes, such as carbocyanins, DiOC_*n*_(3), or Rhodamine 123, accumulate inside polarized cells because viable cells are permeable to those dyes (Díaz et al., 2010; Tracy et al., 2010). Nevertheless, Gram negative bacteria outer membranes can present a barrier to lipophilic dyes uptake. However, a mild treatment with a chelating agent such as Tris-EDTA (ethylenediamine tetraacetic acid) can overcome this limitation (Tracy et al., 2010; Boda et al., 2015).

Anionic and lipophilic dyes, such as those belonging to oxonols family accumulate inside non-viable cells and concentrate by association with intracellular compounds. Without permeabilization protocols, oxonols uptake is more related to membrane integrity rather than to membrane potential and depolarization (Díaz et al., 2010). DiBAC_4_(3) (bis-(1,3-dibarbituric acid)-trimethine oxonol) or BOX seems to be the most used recently to detect depolarized cells of numerous species after antimicrobial treatment (Novo et al., 2000; Wu et al., 2010a,b; Silva et al., 2011; Caldeira et al., 2013; Kramer and Muranyi, 2014; Duarte et al., 2015; Lee et al., 2015; Coronel-León et al., 2016; Grau-Campistany et al., 2016; Kramer and Thielmann, 2016).

#### Metabolic activity

Metabolic activity detection suggests the absence of cell death, but giving the conclusion of alive cell or dead cell is difficult in the case of cell damage, dormancy, and starvation (Nebe-von-Caron et al., 2000; Díaz et al., 2010). In general, a non-fluorescent permeant substrate is taken up by the cell by diffusion and converted inside the cell by intracellular enzymes to a fluorescent substance which is ideally retained in cells with intact membranes (Tracy et al., 2010).

##### Respiratory activity

Bacterial cells with electron transport system activity or respiratory activity are able to reduce 5-cyano-2, 3-ditolyl tetrazolium chloride (CTC) to an insoluble fluorescent CTC-formazan product that accumulates inside the cells (Caldeira et al., 2013; Ferreira et al., 2014; Duarte et al., 2015). For CTC-formazan fluorescence analysis, two regions of CTC-formazan relative fluorescence were analyzed, depending on the fluorescence intensity of positive and negative controls (Ferreira et al., 2014; Morishige et al., 2015). Ferreira et al. (2014) and Díaz et al. (2010) expressed the limit of this stain: CTC staining allows the detection of the most metabolically active bacteria, cells with low respiratory activity may not be detected as CTC-positive, probably due to the relative toxicity of cellular CTC accumulation.

##### Enzymatic activity

Esterase activity is the most common way to evaluate enzymatic activity (Díaz et al., 2010), particularly in studies about damages after antimicrobial treatment (Ananta et al., 2004; Hayouni et al., 2008; Ananta and Knorr, 2009; Schenk et al., 2011; Ayari et al., 2013; Thabet et al., 2013; Antolinos et al., 2014; Kramer and Muranyi, 2014; Surowsky et al., 2014; Hong et al., 2015; Combarros et al., 2016; Kramer and Thielmann, 2016; Meng et al., 2016). Fluorescein and fluorescein derivatives have been used for a wide range of microorganisms as probes for enzymatic activity measurement (Díaz et al., 2010). Among these, carboxyfluorescein diacetate (cFDA) is used primarily for the evaluation of esterase cellular activity (Ananta et al., 2004). It is a lipophilic non-fluorescent precursor that rapidly diffuses across the cell membranes. In the intracellular compartment, diacetate groups of cFDA are hydrolyzed by unspecific esterases into carboxyfluorescein (cF) which is a polar membrane-permeant fluorescent compound. The cells only remain fluorescent if their membranes are intact, thus for cells to be associated as viable, this probe requires both active intracellular enzymes and intact membranes (Hoefel et al., 2003). Moreover, efflux of cF upon glucose addition could also be used as an additional indicator of metabolic performance of the cell (Ananta et al., 2004). Schenk et al. (2011) showed how carefully this staining must be used. Untreated *E. coli, Listeria innocua*, and *Saccharomyces cerevisiae* stained with cFDA showed a heterogeneous behavior in their fluorescence labeling properties. Not all cells yielded high green fluorescence and appeared in expected quadrant in the cytogram. They formulated two hypotheses: (i) the presence of the outer membrane with lipopolysaccharides in Gram negative bacteria or the thick wall of peptidoglycan in Gram positive bacteria which does not allow cFDA freely diffusing across cytoplasmic membrane; (ii) the active expulsion of cF outside the cell by bacteria pumps and consequently the lack of green fluorescence despite the existence of metabolic activity.

Anyway, concentrations of probes, incubation periods, cytometric setting, and control should be assessed for each studied bacteria individually (Kramer and Thielmann, 2016), as performed by Nexmann Jacobsen et al. (1997) for *Listeria monocytogenes*. They compared the use of LIVE/DEAD® BacLight™ kit, Rhodamine 123, 2′,7′-bis(2-carboxyethyl)-5(6)-carboxyfluorescein acetoxymethylester (BCECF-AM), Chemchrome B and cFDA. In conclusion, they determined that only cFDA and Chemchrome B were suitable for rapid, almost real time counting of pure cultures of *L. monocytogenes* by flow cytometry, after 6−24 h incubation in selective enrichment media.

FCM with fluorescent dyes can thus be considered as a suitable tool for the assessment of structural and/or functional microbial cell properties such as metabolic activity, membrane potential, and integrity. The combination of dyes is a pertinent multi-method approach to reveal the presence of intermediate physiological states between life and cell death, showing the heterogeneities of microbial populations. Nebe-von-Caron et al. (2000) classified cells according to some active functions or the integrity of cell structures. These authors distinguished among reproductively viable, metabolically active, intact, and permeabilized cells.

A multi-method approach is a suitable tool to monitor the impact of inactivation treatments on bacteria, providing information about the mode of action, the heterogeneity of populations, species-specific differences to stressors and valuable insight in vital functions beyond pure culturability (Kramer and Thielmann, 2016).

**Figure 1:**
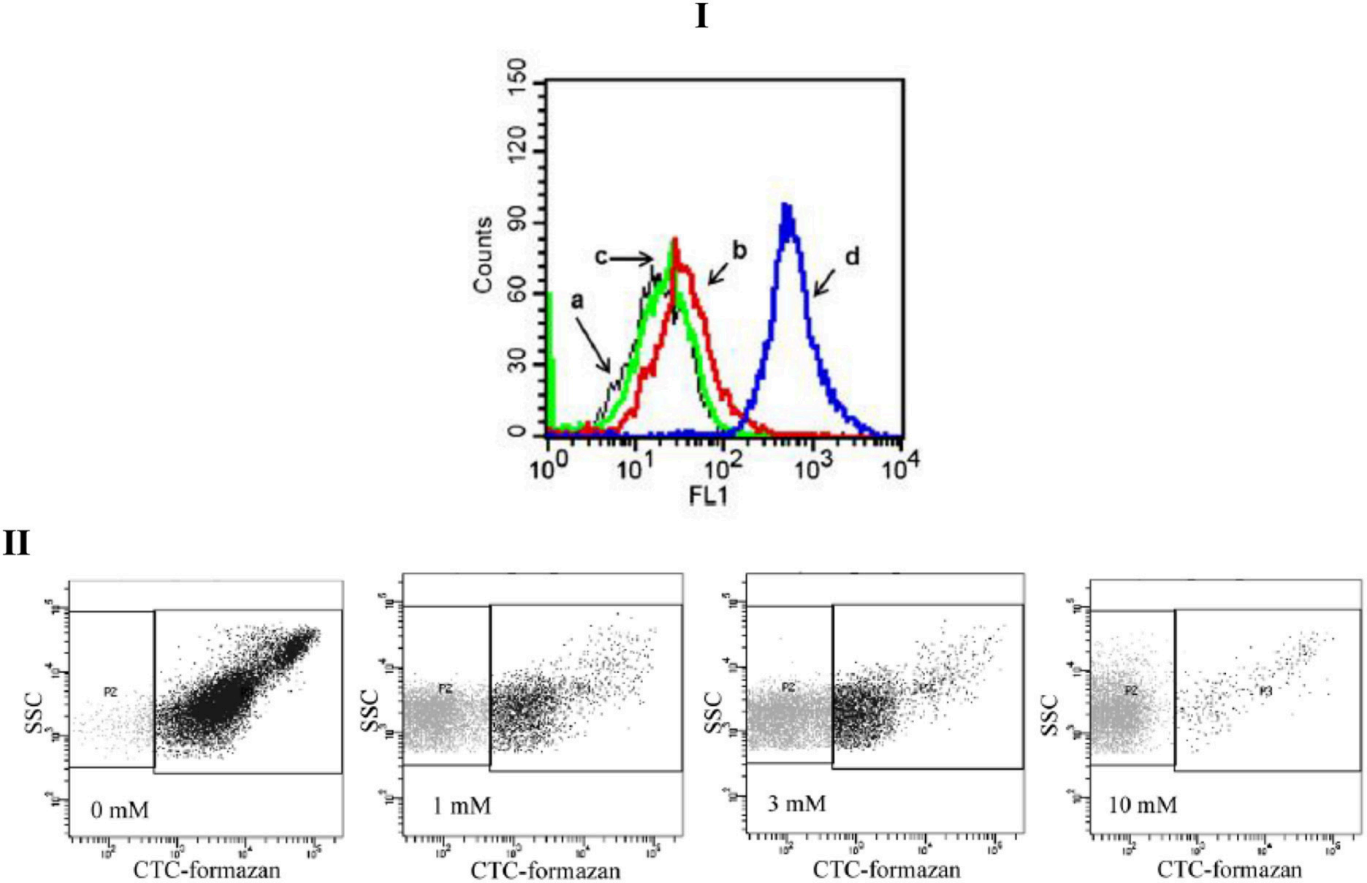
**Examples data acquisition obtained with single dye**. **(I)** Membrane permeabilization of *candida albicans*, detected by SYTOX green fluorescence. The cells were treated with scolopendin 2, BUF (6−21), or Melittin at MIC. (a) Control, (b) Scolopendin, (c) BUF (6−21), (d) melittin (Lee et al., 2015). **(II)** Respiratory Activity of H_2_O_2_–treated *Salmonella entertitids* cells analyzed by flow cytometry: Cytograms of CTC-stained cells. The horizontal axis indicates the fluorescence intensity of CTC-formazan; and the vertical axis, side-light scatter intensity. The respiratory-active Subpopulation was gated in the right rectangle (P3); and the inactive subpopulation, in the left one (P2). H_2_O_2_ concentrations: 0, 1, 3, or 10 mM (Morishige et al., 2015).

The results obtained by FCM are in the form of graphical visualization of scattering and fluorescence cell parameters, which are being analyzed and stored for further analysis (Díaz et al., 2010). Acquired data are identified as events, as the number of cells showing a desired physical property or probe. In the case of single-staining cells, two types of graphical results could be obtained (e.g., Figure 1). A mono-parametric histogram presents the number of cells (*y*-axis) vs. the scattering or fluorescence intensity (*x*-axis; e.g., Figure 1-I). Lee et al. (2015) detected membrane permeabilization of *Candida albicans* by Sytox Green fluorescence. Cells treated by scolopendin 2 and melittin indicated a significant increase in fluorescence intensity. These results show that the fungal cell membranes were permeabilized by the peptides. A bi-parametric histogram represents the intensity of the signals corresponding to different parameters in each axis (e.g., Figure 1-II: scattering intensity (y-axis) vs. CTC-fluorescence activity). Each dot represents a single cell and different regions can be defined into the cytogram to describe cells physiological state.

The publication of Silva et al. (2011) showed well the value of this approach in determining the mechanism of coriander essential oil against *Candida* spp. with three dyes: PI (membrane permeability), DiBAC_4_ (membrane potential), DRAQ5 (DNA staining). Firstly, the percentage of PI-positive cells seemed to depend on essential oil concentration: higher essential oil concentration caused higher membrane permeability. The structure of the cell membrane was disrupted by the essential oil. Permeation to PI, particularly following short incubation period, such as 30 min, indicated that the mode of action of the essential oil involved a lesion of the cell membrane that resulted from direct damage to the cell membrane instead of a metabolic impairment leading to secondary membrane damage. Secondly, the percentage of depolarized cells was also essential oil concentration dependent. Thirdly, DNA distribution histograms were very similar in the control and in essential oil-treated cells, which could indicate that coriander essential oil did not interfere with DNA synthesis. However, at ½Minimal Inhibitory Concentration (MIC), fluorescence intensity values were slightly different: essential oil-treated cells showed higher fluorescence intensity values than control cells. This could indicate that in response to cell damage by coriander essential oil, cells were synthesizing more DNA in order to repair damage functions. Moreover, at 1 MIC, lowest fluorescence intensity values were observed, probably indicating DNA leakage from the cells. In conclusion, these three staining procedures permitted Silva et al. (2011) to propose an antimicrobial mechanism: coriander essential oil killed *Candida* spp. by damaging the cytoplasmic membrane, leading to an impairment of all cellular functions. In the same way, Teng et al. (2014) used two dyes (PI and FITC-labeled AvBD103b) to demonstrate that defensin AvBD103b destroyed the membrane integrity. The antimicrobial target of the defensin was the *Salmonella enteritidis* CVCC3377 cell membrane. A strong permeation occurred in just 5 min treatment. Nevertheless, they made Transmission Electron Microscopy (TEM) observations and followed cellular DNA by spectroscopy to elucidate further the antimicrobial mechanism. Membrane injury was not the only mechanism of microbial killing. In the same way, by using PI-DiBAC4-EB-cFDA individual staining, Kramer and Muranyi (2014) demonstrated that pulsed light exposition induced oxidative stress with concomitant damage to the DNA molecule which were directly responsible for the loss of culturability of *L. innocua* and *E. coli* cells rather than a direct rupture of cell membranes or inactivation of intracellular enzymes.

Multi-parameter FCM analysis allowed highlighting VBNC cells populations such as in the study of Morishige et al. (2015). They treated an environmental isolate of *Salmonella enteritidis* clone (SE C1#15–1) with different concentrations of H_2_O_2_ and analyzed by MP–FCM the respiratory activity, the glucose-uptake activity and DNA synthesis activity individually. Compared with plate count results, they defined VBNC populations as “metabolically active but non-culturable.” H_2_O_2_-treated *S. enteritidi*s cells lost their respiratory activity in a dose-dependent manner. Besides, they showed that H_2_O_2_ treatment did not severely decrease the glucose-uptake activity. These results suggested that metabolic mechanism of glucose-uptake might have been more tolerant to H_2_O_2_treatment than aerobic respiration. Finally, the subpopulation of cells with DNA synthesis disappeared almost entirely after treatment with more than 3 mM H_2_O_2_. Therefore, the higher the concentration of H_2_O_2_ was, the lower the population of cells showing activities of viable cells. Nevertheless, the size of the population varied considerably among the biological activities: glucose-uptake being the largest, CTC-reduction and DNA synthesis, the smallest. In this manner, Morishige et al. (2015) showed that VBNC state might be divided differently according to the metabolic activities by which they were estimated. This illustrated the value of MP-FCM analysis to screen several physiological responses in order to describe thoroughly the cellular state. Moreover, Kramer and Thielmann (2016) revealed the difficulty to discuss with only one staining and the importance of the complementary results of the multi-staining approach. They developed this idea especially for the use of cFDA. Indeed cF, a fluorescent molecule released by esterase activity on cFDA, is usually retained in intact bacteria but leaks from damaged cells, leading to a lower fluorescence intensity. cFDA, esterase substrate may not only serve as an indicator for enzymatic activity but also for membrane integrity. The discrimination of cells with compromised membrane from cells with inactive esterase is difficult. Kramer and Thielmann (2016) recommended to check for a time dependent change in the fluorescence properties of cF-stained cells and also to include a membrane impermeable dye like PI in the characterization of bacterial cells to check membrane integrity. This limitation encouraged many authors to develop a different MP-FCM approach, namely the use of two dyes simultaneously.

### Multi-parameter flow cytometric analysis: simultaneous use of dyes

**Figure 2:**
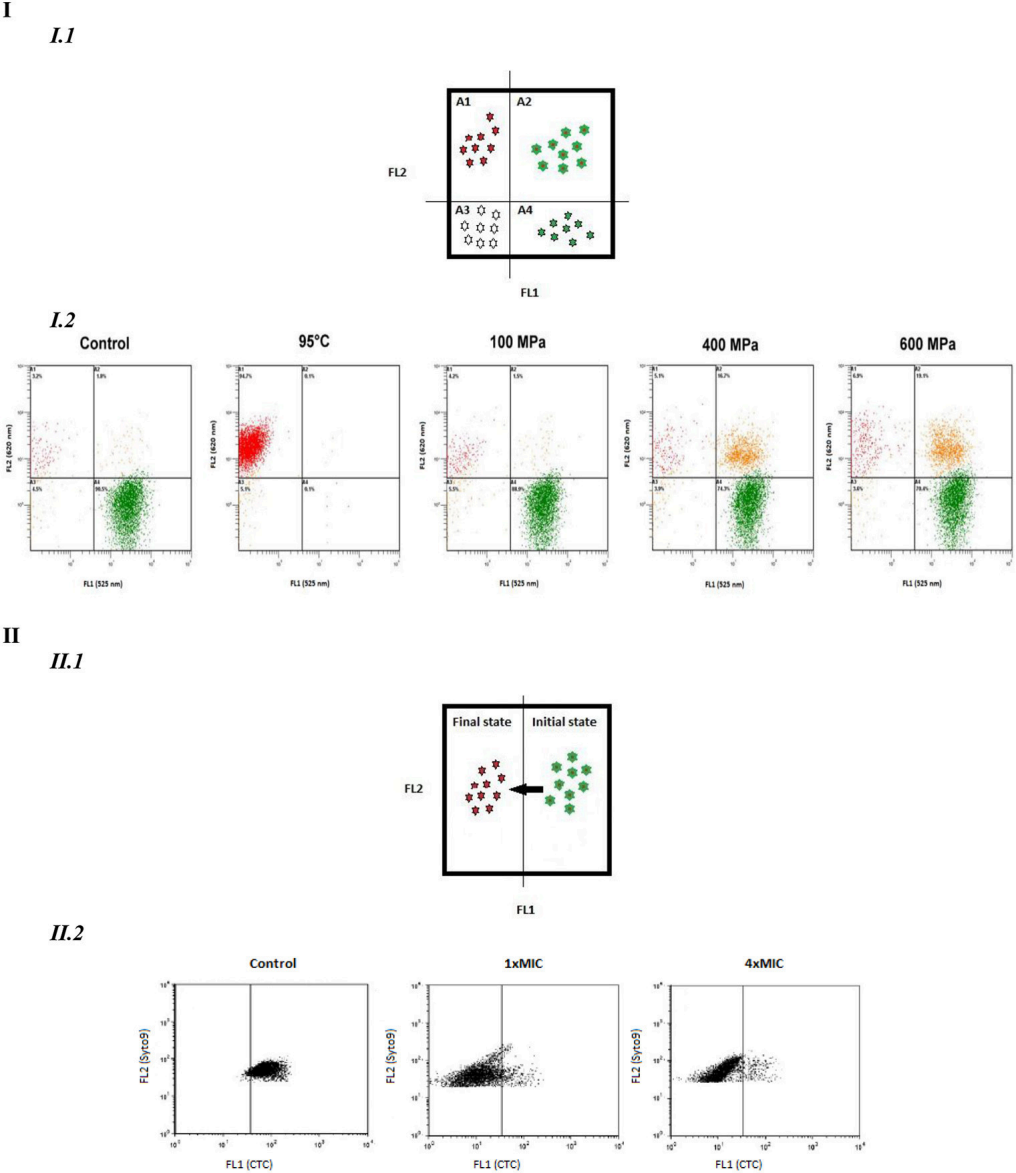
**Dual parameter FCM dot plots**. **(I)**. Dual parameters with two discriminated dyes. I.1. Four different behaviors detected: quadrant A1, FL1−/FL2+; quadrant A2 FL1+/FL2+; quadrant A3, FL1−/FL2−; quadrant A4 FL1+/FL2−. I.2. Dots blots of Lactobacillus rhamnosus GG to assess the effects of different pressure (100,400,600 MPa) and thermal (95°C)treatment on esterase activity (cFDA) and membrane integrity (P1): quadrant A1, cF−/P1+; quadrant A2, cF+/P1+;quadrant A3, cF−/P1−; quadrant A4, cF+/P1− (Ananta et al., 2004). **(II)**. Dual parameter dot plots with one discriminated dye. II. 1 Two different behaviors detected: initial state/Final state. II. 2.Syto9-CTC stained *Arcobacter cryaeophilus* LMG 10829 with resveratrol at concentration of 0 (control), 1xMIC and 4xMIC: initial state: Syto9+/CTC; Final state: Syto9+/CTC−(Ferreira et al., 2014).

Simultaneous double-staining could allow characterizing an intermediate state where cells show fluorescence with both probes (Figure 2). The analysis with several dyes depends on the compatibility of the dyes namely spectral emission and staining conditions. If the two dyes are discriminating, four quadrants can be delimited corresponding to four different physiological states (Figure 2-I). If only one dye is discriminating, such as Syto9® which is able to enter all cells, two states are described: an initial and a final state (Figure 2-II).

Different dye couples can be used depending on the targeted cell functions (Table 2).

**Table 2:** **Multi-fluorescence properties of double-stained cells collected in a same area with the explanation of the underlying cellular mechanism**.

	**Dyes**	**Fluorescence**	**Multi-fluorescence**		**Status of cellular mechanism**
I. Membrane integrity	Syto9 (or SYBR-I, Syto13)/PI	Green/Red	Syto9+	PI−	Intact cells 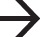 Viable cells
			Syto9−	PI+	Permeabilized cells 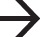 Dead cells
			Syto9+	PI+	Partially-permeabilized cells 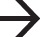 Injured cell populations
			Syto9−	PI−	Unstained debris
	References: Possemiers et al., 2005; Pianetti et al., 2008; Kim et al., 2009; Muñoz et al., 2009; Spilimbergo et al., 2010; Martínez-Abad et al., 2012; Choi et al., 2013; Tamburini et al., 2013; Booyens and Thantsha, 2014; Fernandes et al., 2014; Manoil et al., 2014; Pal and Srivastava, 2014; Tamburini et al., 2014; Boda et al., 2015; Ferrentino et al., 2015; Freire et al., 2015; Li H. et al., 2015; Li W. et al., 2015; Muriel-Galet et al., 2015				
	TO/PI	Green/Red	TO+	PI−	Intact cell membranes
			TO−	PI+	Permeabilized cell membrane
			TO+	PI+	Cell membrane damaged, slightly permeabilized
			TO−	PI−	Damage of DNA and RNA while the cell may still be intact
	References: Surowsky et al., 2014				
II. Membrane integrity/physiological state					
A. Enzyme activity	PI/cFDA	Red/Green	PI+	cF−	Esterase activity not detectable, membrane compromised 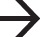 dead cells
			PI+	cF+	Viable injured or stressed cells with intracellular activity and minimally compromised membrane
			PI−	cF+	Active esterase, intact membrane 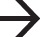 viable cells
			PI−	cF−	Inactive esterase or cF extruded out of the cells, intact membrane, or unstained debris
	References: Ananta et al., 2004; Hayouni et al., 2008; Ananta and Knorr, 2009; Schenk et al., 2011; Ayari et al., 2013; Thabet et al., 2013; Surowsky et al., 2014; Hong et al., 2015; Combarros et al., 2016; Meng et al., 2016				
B. Pump activity	Syto9/EB	Green/Red	(Syto9+ EB+ green and red high intensity)		Cells with efflux pump completely damaged
	References: Kim et al., 2009				
C. Membrane potential	IP/DiBAC_4_	Red/Green	PI−	DiBAC_4_−	Unstained population of intact cells
			PI−	DiBAC_4_+	Depolarized cells
			PI+ PI+	DiBAC_4_− DiBAC_4_+	Population of permeabilized cells with different degrees of damage
	References: Novo et al., 2000; Wu et al., 2010a,b; Coronel-León et al., 2016; Grau-Campistany et al., 2016				
	Syto40/DiBAC_4_	Blue/Green	Syto40+	DiBAC_4_+	Depolarized cells
			(blue and green high intensity)		
	References: Duarte et al., 2015				
	TO-PRO®3/DiOC_2_(3)	Far red/Green shift to red	TO-PRO®3+	DiOC_2_(3)+	Permeabilized and depolarized
			TO-PRO®3+	DiOC_2_(3)−	Permeabilized and polarized
			TO-PRO®3−	DiOC_2_(3)+	Non-permeabilized and depolarized
			TO-PRO®3−	DiOC_2_(3)−	Non-permeabilized and polarized
	References: Novo et al., 2000; Hammer and Heel, 2012				
D. Respiratory activity	Syto40/CTC	Blue/red	Syto40+	CTC+	Cells with respiratory activity
			(blue and red high intensity)		
	References: Duarte et al., 2015				
	Syto9/CTC	Green/red	Syto9+	CTC+	Cells with respiratory activity
			(green and red high intensity)		
	References: Ferreira et al., 2014				
III. DNA/Antibody β-lactamase activity	Syto62/anti β -lactamase mAb AF488-DAM IgG	Red/Green	Syto62+	anti β-lactamase mAb AF488-DAM IgG+	Cells with β-lactamase activity 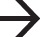 ampicillin resistant
	References: Huang et al., 2016				

*PI, Propidium Iodide; TO, Thiazole Orange; cFDA, carboxyFluorescein DiAcetate; DiBAC_4_, Bis-(1,3-DibutylBarbituric ACid) Trimethine Oxonol = bis-oxonol = BOX; DiCO_2_, 3,3-DiethyloxaCarbocyanine Oxide; CTC, 5-Cyano-2,3-ditolyl Tetrazolium Chloride; EB, Ethidium Bromide*.

#### Membrane integrity

Using the combination between a Syto family compound and PI, four populations could be distinguished corresponding to live, dead, injured cells, or unstained debris (Possemiers et al., 2005; Kim et al., 2009; Muñoz et al., 2009; Martínez-Abad et al., 2012; Choi et al., 2013; Booyens and Thantsha, 2014; Fernandes et al., 2014; Manoil et al., 2014; Pal and Srivastava, 2014; Boda et al., 2015; Freire et al., 2015; Li H. et al., 2015; Li W. et al., 2015). The quantification of intact cells by FCM was based on membrane integrity, which is a more general property that demonstrates the protection of cell constituents and potentially capable of metabolic activity/repair and reproductive growth (Spilimbergo et al., 2010).

During an antimicrobial treatment, an increase of unstained population could appear as reported by Booyens and Thantsha (2014). They formulated two hypotheses: (i) a correspondence to the cells that have undergone severe lysis and thus lost their nucleic acids, thereby rendering them unstainable called “ghost cells”; (ii) cells that clumped together or formed interlaced chains which may decrease staining accuracy, correlated to a change in light scatter signals.

Thiazole orange (TO) is another dye for membrane integrity, which is very interesting because it is able to bind both to DNA and RNA. Since TO-DNA complex has a higher fluorescent intensity than TO-RNA complex, this allows a differentiation. Based on this approach, Surowsky et al. (2014) defined thus the bactericidal action of cold plasma against *Citrobacter freundii* as a mechanism based on cell permeabilization and RNA damage.

#### Membrane integrity/physiological state

Enzymatic activity is principally studied by using cFDA dye (Table 2). After treatment, four kinds of subpopulations were observed with different staining characteristics: (i) lower left quadrant, unstained debris; (ii) upper left quadrant, PI-labeled cells considered as dead; (iii) lower right quadrant, cF-labeled cells considered as viable; (iv) upper right quadrant, double stained population considered as injured/compromised (Hayouni et al., 2008; Ayari et al., 2013). In Hayouni et al. (2008) study, the percentage of the unstained subpopulation after treatment with essential oils did not exceed 8%. Lactobacilli are well-known to exhibit a planar division with cells remaining attached to each other, thus producing chains characteristic for the genus and misleading FCM results. This phenomenon was avoided in many works by using sonication treatment of the cells or gentle agitation (Hayouni et al., 2008). With a PI/cFDA staining, Hong et al. (2015) explored the antimicrobial mechanism of an antimicrobial peptide, Tachyplesin I, against *E. coli* and *Staphylococcus aureus* cells. The fraction of *E. coli* cells with PI fluorescence increased proportionally with increase in the concentration of Tachyplesin I, as well as the fraction with cF fluorescence decreased. Nevertheless, intracellular esterase inactivation and membrane damage did not occur simultaneously after exposure to a low concentration of Tachyplesin I (5 μg.mL^−1^), whereas bacteria died instantaneously at concentrations exceeding 5 μg.mL^−1^. They detected injured bacteria which cannot form visible colonies on agar plates, but which exhibited detectable metabolic activity. Schenk et al. (2011) highlighted difference of strains behavior after exposure to thermal treatment. For *E. coli* and *S. cerevisiae*, inactivation coincided with compromise of membrane, whereas for *L. innocua*, four subpopulations appeared. For this latter strain, thermal treatment induced cell death and was achieved in presence or absence of membrane degradation and in cells with or without enzymatic activity depending on the intensity of the treatment.

The dual staining with a cell-permeant dye and a dye detecting loss in membrane potential is another interesting possibility to distinguish between different degrees of damage. Coronel-León et al. (2016) used PI-DiBAC_4_ double-staining to study the antimicrobial mechanism of N^α^-lauroyl arginate ethylester (LAE®). The reduction in viability of *Yersinia enterocolitica* was evidenced for sub-MIC values of LAE®, the bactericidal action of LAE® increased with its concentration. Even if a similar effect was observed on *Lactobacillus plantarum*, a high proportion of unstained population was found despite the high reduction in viability. This suggested that the effect of LAE® on *L. plantarum* not only caused membrane depolarization and permeability but also some other non-specific effects inside the cell, such as a collapse of the cytoplasmic material, could be observed. Novo et al. (2000) reported a discrepancy between the drop in membrane potential of the cytoplasmic material and cell viability, suggesting a recovery in the bacterial population after the initial antimicrobial impact. Nucleic acid dyes should be used with caution as an indicator of cell death (Novo et al., 2000). This was the case of LAE®-treated *L. plantarum*: 72% of the population remained unstained, while the reduction in viability was 98.6%. This difficulty could be due to the multi-target sites of biocides, or to a reversion to an impermeable state (Novo et al., 2000). This illustrated well the need to analyze several parameters to have a comprehensive understanding of the mechanism (Coronel-León et al., 2016).

Novo et al. (2000) compared two couples of dyes, PI/DiBAC_4_ and TO-PRO®3/DiOC_2_, to analyze antibiotic effects on membrane potential, membrane permeability, and bacterial counts of *S. aureus* and *Micrococcus luteus*. The results for membrane permeability were comparable with PI and TO-PRO®3 staining, whereas discrepancies were measured between the results obtained with DiBAC_4_ and DiOC_2_for measurement of membrane permeability. Novo et al. (2000) expressed reservation about DiBAC_4_staining which was strongly influenced by cell size.

Another element to take into account is the nature of microorganisms: the behaviors of yeast and bacterial cells may differ. Thabet et al. (2013) treated *S. cerevisiae* cells by UV-A photocatalysis. Damaged cells, PI- and cF-fluorescent cells, were culturable. This was probably due to the greater complexity of cell organization compared to bacteria. Damages to the membrane do not imply an immediate enzyme activity break and cell death. For example, contrary to bacteria, respiration functions are not supported by plasma membrane, but in yeast and other eukaryotic organisms, they are compartmented to mitochondria. Thus, considering exclusively the membrane status to characterize photocatalysis effects on yeast cell viability appeared to be insufficient (Thabet et al., 2013).

Following analysis by combining dyes, a single staining may also be done in addition to not excessively complicate the analysis. This is often the case for the screening of depolarization by DiBAC_4_ or DiOC_2_. In several works, these dyes were used to study the polarization of cell membrane in parallel of a double-staining experimentation (Pianetti et al., 2008; Surowsky et al., 2014; Tamburini et al., 2014; Li W. et al., 2015). (Li W. et al., 2015) performed a Syto9/PI double-staining and a single DiOC_2_ staining of *E. coli* cells to study the antimicrobial mechanisms of proline-rich peptides (monomers, dimers, and tetramers). The shift in the green fluorescent population as peptide concentration increased, indicated a shift from a mixed hyperpolarized and depolarized cell population to a more depolarized membrane population. With all MP-FCM results, they concluded that multimerization of the Chex-Arg20 monomer to dimer and tetramer altered the mode of action from non-lytic to a membrane disruptive capacity.

#### DNA/antibody

Huang et al. (2016) reported the development of a high sensitive FCM method to probe minority population of antibiotic-resistant bacteria. Nucleic acid dye Syto62 was used to stain all the bacteria red. Then, monoclonal antibody against TEM-1 β-lactamase and Alexa Fluor 488-conjugated secondary antibody were used to selectively label resistant bacteria which retained β-lactamase activity green. This immunofluorescent-staining method remains marginal as it requires specific antibodies to be available.

Fernandes et al. (2014) expressed reservations about the use of MP-FCM. Intermediate states are generally misclassified or simply identified as “unknown” and poorly characterized population. According to them, a way to circumvent this limitation is through a well-defined gating strategy that is highly dependent on how accurate the positive and negative controls are in order to define cell population (viable, compromised, and dead cells). Moreover, that is also why other methods could be applied in parallel for a better description of the cell populations identified by MP-FCM (Table 3).

**Table 3:** **Additional methods to complete MP-FCM analysis in order to better describe cell populations after exposure to an antimicrobial treatment**.

**Additional method References**	
**MICROSCOPY**	
Transmission Electron Microscopy (TEM)	Ayari et al., 2013
	Choi et al., 2013
	Coronel-León et al., 2016
	Hong et al., 2015
	Li H. et al., 2015
	Teng et al., 2014
	Wu et al., 2010a
Scanning Electron Microscopy (SEM)	Ferreira et al., 2014
	Li H. et al., 2015
	Hong et al., 2015
	Muriel-Galet et al., 2015
	Spilimbergo et al., 2010
	Surowsky et al., 2014
Fluorescence microscopy	Fernandes et al., 2014
	Hong et al., 2015
	Li W. et al., 2015
	Tamburini et al., 2013
	Thabet et al., 2013
**SPECTROSCOPY**	
Fourier Transform infrared spectroscopy (FTIR)	Booyens and Thantsha, 2014
	Meng et al., 2016
Nuclear Magnetic Resonance spectroscopy (NMR)	Tamburini et al., 2014
Circular Dichroism spectroscopy (CD)	Teng et al., 2014
Membrane models	Lee et al., 2015
	Grau-Campistany et al., 2016
	Wu et al., 2010a
Propidium MonoAzide quantitative-Polymerase	Ferrentino et al., 2015
Chain reaction (PMA-qPCR)	Tamburini et al., 2013

## Correlation with plate counts and other techniques used simultaneously

### Viability vs. culturability

Nowadays it is accepted that a cell population exposed to a stress can cause the appearance of different cell populations and, in particular sub-lethally stressed/injured cells which could be defined as “active but non-culturable” or “viable but non-culturable.” Nevertheless, the definition of these states is still controversial (Antolinos et al., 2014). The presence of such injured bacteria in food or in clinical applications might be critical in terms of their potential activity on excreting toxic or food spoiling metabolites, on transferring of genes (Ananta et al., 2004; Schenk et al., 2011; Ayari et al., 2013; Hong et al., 2015). Even if FCM analysis provides high-speed information at real time on damage at single cell level, plate count method also gives an indication of cells able to grow at a certain time.

First of all, Kramer and Thielmann (2016) described bacterial cell aggregation after a heat treatment. They discussed that this phenomenon could lead to an erroneous result and an overestimation of the inactivation. Indeed a single colony on the plate could contain a cell aggregate, namely more than one culturable cell (Hayouni et al., 2008). Agglomeration effect remains undetected with classical culture-based methods, then FCM acquisition was in this case a very useful method (Manoil et al., 2014).

Most studies included fluorescence staining FCM analysis and plate counting to monitor antimicrobial effects. Nevertheless, the consistency of results obtained by these two methods varied greatly across studies. On one hand, some revealed a good correlation; while on the other hand, others showed clearly a significant discrepancy between conventional plate counts and different viability staining parameters (Table 4). If the comparison between Colonies Forming Units (CFU) and FCM results shows a significant difference, this suggests the presence of sub-lethally stressed subpopulations, not able to form colonies on agar plates (Ayari et al., 2013). Such observations depended on the nature of antimicrobial treatments applied, and thus likely on underlying mechanisms involved. These studies also revealed differences of behavior between strains for the same treatment. The question whether injured cells are able or not to retain their proliferation capacity when returned to a favorable environment is still under debate (Manoil et al., 2014).

**Table 4:** **CFU and MP-FCM results correlation and non-correlation**.

	**Antimicrobial treatment**	**Target microorganisms**	**MP-FCM Dyes**	**Reference**
CFU and FCM results correlation	Carvacrol + nisin combined with irradiation	*Bacillus cereus* (vegetative cells)	PI/cFDA	Ayari et al., 2013
	Pulsed magnetic fields	*Staphylococcus* spp., *Escherichia coli*	Syto9/PI	Boda et al., 2015
	TiO_2_-nanoparticles	*Pseudomonas putida*	SYBR-I, PI/cFDA	Combarros et al., 2016
	Lipopeptide antibiotics derived from polymyxin B	*Staphylococcus aureus, E. coli*	PI/DiBAC_4_	Grau-Campistany et al., 2016
	Essential oils	Lactic acid bacteria	PI/cFDA	Hayouni et al., 2008
	Essential oils	*Listeria monocytogenes*	Syto9/PI	Muñoz et al., 2009
	D-*erythro*-sphingosine	*Escherichia coli*	Syto9/PI	Possemiers et al., 2005
	UV-A photocalalysis	*Saccharomyces cerevisiae*	PI/cFDA-AM	Thabet et al., 2013
CFU and FCM results non-correlation	High hydrostatic pressure	*Lactobacillus rhamnosus* GG	PI/cFDA	Ananta et al., 2004
	Carvacrol or nisin	*Bacillus cereus* (vegetative cells)	PI/cFDA	Ayari et al., 2013
	Resveratrol inclusion complexes	*Campylobacter* spp., *Arcobacter butzleri*	Syto40/DiBAC_4_	Duarte et al., 2015
	Super-critical CO_2_	*Listeria monocytogenes*	SYBR-I/PI	Ferrentino et al., 2015
	Super-critical CO_2_	*Salmonella enterica*	Syto9/EB	Kim et al., 2009
	Pulsed light	*Listeria innocua, E. coli*	PI, DiBAC_4_, EB, cFDA	Kramer and Muranyi, 2014
	Thermal treatment	*L. innocua, E. coli, S. aureus, S. enterica*	PI, DiBAC_4_, EB, cFDA	Kramer and Thielmann, 2016
	Super-critical CO_2_	*S. aureus, E. coli*	Syto9/PI	Li H. et al., 2015
	Blue light-activated curcumin	*Streptococcus mutans*	Syto9/PI	Manoil et al., 2014
	Silver	*L. monocytogenes, S. enterica*	Syto9/PI	Martínez-Abad et al., 2012
	Ultra-high hydrostatic pressure and mild heat	*Bacillus subtilis* (vegetative cells)	PI/cFDA	Meng et al., 2016
	NaCl	*Aeromonas hydrophila*	SYBR-I/PI	Pianetti et al., 2008
	UV-C light	*L. innocua, E. coli, S. cerevisiae*	PI/cFDA	Schenk et al., 2011
	Super-critical CO_2_	*S. cerevisiae*	SYBR-I/PI	Spilimbergo et al., 2010
	Super-critical CO_2_	*L. monocytogenes, S. enterica, E. coli*	SYBR-I/PI	Tamburini et al., 2013

*PI, Propidium Iodide; cFDA, carboxyFluorescein DiAcetate; cFDA-AM, carboxyFluorescein DiAcetate-acetoxymethyl; DiBAC_4_, Bis-(1,3-DibutylBarbituric ACid) Trimethine Oxonol = bis-oxonol = BOX; EB, Ethidium Bromide*.

#### Viability-culturability correlation

On one hand, some researchers showed a strong correlation between MP-FCM and plate counting method results (Possemiers et al., 2005; Hayouni et al., 2008; Muñoz et al., 2009; Ayari et al., 2013; Thabet et al., 2013; Boda et al., 2015; Combarros et al., 2016; Grau-Campistany et al., 2016). For example, Muñoz et al. (2009) used MP-FCM and plate counting to evaluate the viability of *L. monocytogenes* exposed to several essential oils with antibacterial properties. By comparing MP-FCM results and plate counts, they assumed that compromised cells may recover and grow in appropriate media. As well, Live/Dead analysis proved to be a good alternative to plate counts to monitor the antimicrobial effect of D-*erythro*-sphingosine in saline toward three intestinal pathogens (Possemiers et al., 2005). According to Grau-Campistany et al. (2016), the viability reduction obtained by plate count is generally in good correlation with membrane damage caused by antimicrobial peptides.

#### Absence of correlation between viability and culturability

One the other hand, for example, carbon dioxide treatment seemed to be a treatment causing the appearance of VBNC cells (Kim et al., 2009; Spilimbergo et al., 2010; Tamburini et al., 2013; Ferrentino et al., 2015; Li H. et al., 2015). After 5 min Super-Critical carbon dioxide (SC-CO_2_) pasteurization treatment at 45 and 50°C of *Listeria* cells, about 4 and 6 log reductions were measured by plate counts, respectively, while the reductions measured by MP-FCM were 2 log at 45°C and 4 log at 50°C (Ferrentino et al., 2015). These results indicated that a substantial amount of *Listeria* cells of about 10^2^ cells.g^−1^ remained intact but non-culturable after treatment. Spilimbergo et al. (2010) performed a study on *S. cerevisiaie* cells in saline solutions treated by SC-CO_2_ at 36°C and 10 mPa and they also indicated that conventional cultivation-based methods did not allow an accurate quantification of intact cells. For a treatment at 5000 rpm-100 bar-36°C during 10 min, the difference was particularly evident with a difference in the percentage of cell survival exceeding 55%. These data indicated a high presence of non-culturable but still intact yeast cells in the treated samples. Nevertheless, after a treatment under the same operative conditions but during 20 min, the difference decreased down to 10% between the two analytical methods. This experimental evidence was probably due to the mechanical irreversible stresses to the cells membranes induced by a longer time. Similar findings were reported on FCM and plate counts analysis to evaluate the effect of SC-CO_2_ (Tamburini et al., 2013) and high-pressure carbon dioxide on food borne bacteria *L. monocytogenes, Salmonella enterica*, and *E. coli* (Garcia-Gonzalez et al., 2010). Conversely, the efflux pump was completely damaged or malfunctioning before the effect of SC-CO_2_ on the culturability of *S. enterica* serotype Typhimurium cells became apparent (Kim et al., 2009).

Kramer and Muranyi (2014) also observed a significant discrepancy between conventional plate counts and different viability staining parameters, which showed that a pulsed light treatment against *L. innocua* DSM 20649 and *E. coli* DSM 498 did not cause an immediate shutdown of vitality functions even when the number of colony-forming units already decreased for more than 6 log_10_ sample^−1^. Even if *L. innocua* DSM 20649 and *S. aureus* DSM 346 showed a same trend of concomitant depolarization and loss of respiration activity after a thermal treatment, colony counts were decreased only by less than 0.5 log for *L. innocua* whereas by more than 3 log for *S. aureus* (Kramer and Thielmann, 2016). A part of the heat induced de-energized *L. innocua* population was still able to recover and multiply, and an opposite trend was observed for *S. aureus*. These results highlighted residual activity of non-culturable bacteria and the strain-dependence of the response to an antimicrobial treatment. In the same study, these authors also observed a strong difference for the uptake of PI after thermal treatment for Gram negative bacteria, which were much more susceptible than Gram positive ones. However, this difference was not reflected by the plate count results: this suggested thus that membrane disruption is not the primary mechanism of mild thermal inactivation of bacterial cells (Kramer and Thielmann, 2016). Even if MP-FCM is a very valuable rapid microbiological method for the assessment of functional properties of bacteria exposed to external stressors, classical plate count method can bring crucial additional information. In fact, reproductive growth requires stringent conditions including both metabolic activity and membrane integrity (Spilimbergo et al., 2010). Manoil et al. (2014) isolated by fluorescent-activated cell sorting an unknown injured population of *Streptococcus mutans* cells which were exposed to blue light-activated curcumin. Then they cultured this subpopulation on agar plates. They indicated that only 0.6% of this unknown injured population was able to grow, thus supporting the idea of injured cells losing their proliferation capacity in their case. Ananta et al. (2004) also showed that the occurrence of esterase activity did not correlate with viability according to plate enumeration. High hydrostratic pressure treated *Lactobacillus rhamnosus* GG cells still possessed residual esterase activity. This metabolic activity was not critical for the maintenance of viability.

The existence of differences between culturability and viability with silver as antimicrobial has already been reported (Martínez-Abad et al., 2012). But, conversely, with silver as antimicrobial, it was found that after a period where viable counts were not detected, bacterial populations recovered, and were able to proliferate in most cases. The resuscitation of the cultures was explained by both the existence of a resilient fraction of bacteria in a compromised state and the parallel inactivation of the silver species (Martínez-Abad et al., 2012).

Ayari et al. (2013) described the two cases (correlation and non-correlation) in the same study. For an intensive treatment, such as *Bacillus cereus* cells pre-treated with carvacrol combined with nisin and then exposed to sub-lethal radiation treatment (1 kGy), CFU results were in accordance with FCM analysis, whereas for single treatment with carvacrol and nisin, good agreement was not found. These results showed that the culturability of subpopulations with identical fluorescence characteristics depends on the treatments imposed to the cells. Therefore, a rapid loss of culturability is not necessarily correlated to the complete cell death (Ayari et al., 2013). In the same way, Hayouni et al. (2008) showed a high correlation between CFU and FCM results, except for *Lactobacillus pentosu*s and *Lactobacillus rhamnosus*. This could be due to the distribution of dead cells in chains.

The presence of VBNC subpopulations was often as a function of the intensity of the treatment. For example, thermal treatments at different temperatures against *L. rhamnosus* resulted in different responses of the cell to PI/cFDA labeling (Ananta and Knorr, 2009). Exposure to 60°C reduced considerably the cF-accumulation activity, however, there was no significant loss of membrane integrity. In contrast, when cells were subjected to 75°C, PI uptake already occurred in the first 90 s. From this staining behavior, they concluded that at higher temperatures the primary target of lethal effect of heat was the bacterial cytoplasmic membrane.

Moreover the correlation seemed to depend on the physiological mechanism which is analyzed. Duarte et al. (2015) showed a lack of direct correlation between the percentage of depolarized cells and the time-kill curves results for *Campylobacter* spp. and *Arcobacter butzleri* after exposure to resveratrol inclusion complexes. The cell membrane depolarization could be only a transition state which would occur before membrane permeabilization (Hammer and Heel, 2012) and might be caused by several factors or antimicrobial agents. The cells in this state would have the capacity to regain culturability. Metabolic activity reduction may not be directly related to a decrease in the number of culturable cells (Ferreira et al., 2014).

### Microscopy

Transmission Electron Microscopy (TEM; Wu et al., 2010a; Ayari et al., 2013; Choi et al., 2013; Teng et al., 2014; Hong et al., 2015; Li H. et al., 2015; Coronel-León et al., 2016), Scanning Electron Microscopy (SEM; Spilimbergo et al., 2010; Ferreira et al., 2014; Surowsky et al., 2014; Hong et al., 2015; Li H. et al., 2015; Muriel-Galet et al., 2015) and fluorescence microscopy (Tamburini et al., 2013; Thabet et al., 2013; Fernandes et al., 2014; Hong et al., 2015; Li W. et al., 2015) could be used to observe cell interior structure, cell surface morphology and localize fluorescence compounds or components, respectively.

Boda et al. (2015) confirmed Syto9/PI double-staining FCM results with TEM micrographs showing the ruptures of *E. coli* cell walls following their exposure to a 4 Tesla pulsed magnetic field.

Spilimbergo et al. (2010) used scanning electron microscopy (SEM) to confirm a clear modification of *S. cerevisiae* cell structure due to CO_2_ treatment. Untreated sample showed cells having a round shape with a smooth surface and no spot, while almost the totality of the cells of the treated sample presented an irregular shape with some dark points/zones, indicating a modification of the cell wall. As well, Muriel-Galet et al. (2015) observed *L. monocytogenes* and *E. coli* cell membranes by SEM: they concluded that the membrane of these bacteria is the main target of LAE®. Microscopic observations allow thus to gain further insights into the inactivation mechanism.

If the studied antimicrobial molecule can be labeled, microscopy methods can also be applied to visualize these molecules on bacterial cells as recently performed by (Li W. et al., 2015). Together with MP-FCM results, these authors could conclude that multimerization of the Chex-Arg20 antimicrobial monomer peptide to dimer and tetramer altered its mode of action against *E. coli* cells from non-lytic to a membrane disruptive capacity. To localize peptides in *E. coli* cells, Alexa-Fluor 647-labeled peptides were assembled and *E. coli* membranes were labeled with FM lipophilic styryl dye (FM, 4-64 FX). With high-resolution fluorescence microscopy, they observed that peptides localized primarily in the cytosol, while at higher peptide concentrations, peptides associated with both the cytosol, and membrane. It was clear that membrane interaction of the dimer and the tetramer induced membrane lysis, whereas the monomer did not.

### Other techniques

#### Spectroscopy

Fourier transform infrared spectroscopy (FTIR) permits to study the entire molecular composition of microbial cells, to reveal the biochemical composition of cellular constituents such as cell wall, membrane (phospholipid bilayer, peptidoglycan, lipopolysaccharides), and cytoplasm (fatty acids, water, nucleic acids, proteins, polysaccharides). Booyens and Thantsha (2014) observed a change in size and granularity of *Bifidobacterium* populations exposed to garlic clove extract by MP-FCM and completed this approach by using FTIR spectroscopy. In the presence of garlic, there was a decrease in lipid content of the membrane, which was an additional element that could best describe the state of the cells and the antimicrobial mechanism. Meng et al. (2016) revealed a change of the membrane phospholipid molecules of *B. subtilis* after an ultra-high hydrostatic pressure and a mild heat (HPMH) treatment by FTIR spectroscopy. They passed from a liquid crystalline state to a gel state with a decrease in membrane fluidity. HPMH treatment decreased the α-helix content, while it increased the random coil content of the cellular proteins, which resulted in protein denaturation.

Tamburini et al. (2014) used the Nuclear Magnetic Resonance spectroscopy (NMR) to observe the changes in cell membrane lipid composition. Firstly, they established the phospholipid profile of *E. coli* K12 membranes and after SC-CO_2_ treatment, they revealed that there were strong perturbations of membrane architecture namely on the two dominant phospholipid species (phosphatidyglycerol and phosphatidylethanolamine). This treatment had no detectable effect on cell density or granularity, whereas the cellular volume changed.

Circular Dichroism spectroscopy (CD) is used extensively to study chiral molecules particularly secondary structure or conformation of proteins sensitive to the environment, temperature, pH modifications. CD can be used to observe how secondary structure of proteins changes with environmental conditions or on interaction with other molecules. Teng et al. (2014) investigated change of the secondary structure of *S. enteritidis* genomic DNA after exposure to the avian defensin AvBD103b. *S. enteritidis* genomic DNA showed a typical negative peak and a positive peak around 240 and 270 nm, respectively. When the cells were treated with AvBD103b, the intensity of the DNA ellipticity became weaker. These results suggested that AvBD103b interacted with the *S. enteritidis* genomic DNA by changing the DNA conformation. With a gel retardation assay, they showed the insertion of the base pairs.

#### Vesicles as membrane models

Lee et al. (2015) elucidated the scolopendin 2 antimicrobial mechanism as a membrane-active mechanism leading to the formation of pores in microbial plasma membrane. Using giant unilamellar vesicles encapsulating calcein and large unilamellar vesicles containing fluorescein isothiocyanate-dextran, which were similar in composition to typical *E. coli* O157:H7 and *Candida albicans* membranes, they demonstrated that scolopendin 2 disrupted membranes, resulting in a pore size between 4.8 and 5.0 nm. Membrane modeling appeared thus as a useful method to corroborate and complete the results obtained by flow cytometry. Grau-Campistany et al. (2016) and Wu et al. (2010a) also used lipid vesicles models of POPG (1-palmitoyl-2-oleoyl-glycero-*sn*-glycero-3-phospho-(1′-rac-glycerol)) or POPE/POPG (1-palmitoyl-2-oleoyl-*sn*-glycero-3-phosphoethanolamine/POPG) to mimic the Gram positive and Gram negative bacterial membrane. Grau-Campistany et al. (2016) correlated MP-FCM data with biophysical experiments in model membranes, with more permeabilization for *E. coli* than for *S. aureus*, corresponding to more leakage from POPE/POPG vesicles than pure anionic POPG vesicles at the same antimicrobial lipopeptide antibiotics concentration. Wu et al. (2010a) described S-thanantin antimicrobial mechanism against *E. coli* and *Bacillus subtilis*: first, it disrupted the membrane permeability, and then depolarized the cell membrane.

#### Propidium monoazide quantitative-polymerase chain reaction (PMA-qPCR)

Propidium monoazide quantitative-Polymerase Chain Reaction (PMA-qPCR) is another viability test method that Ferrentino et al. (2015) used in parallel of a SYBR-PI double-staining. The apparent minor inactivation efficiency evaluated by PMA-qPCR (about 2 log reduction) compared to MP-FCM could be due to incomplete exclusion of dead cells signals leading to false-positive signals, a known drawback of this technique (Ferrentino et al., 2015). The suppression of dead cells signals depends on the complexity of the sample matrix and on the length of DNA amplicon. Besides, PMA-qPCR is also the most sensitive quantification technique compared to MP-FCM and plate counts (Ferrentino et al., 2015). Tamburini et al. (2013) also used PMA-qPCR to assess the effect of SC-CO_2_ treatment on *L. monocytogenes, E. coli*, and *S. enterica* cells, and their PMA-qPCR and FCM results strongly correlated, even if they detected VBNC population. Indeed, a vast majority of cells remained in the partially permeabilized state.

## Conclusion

To investigate the efficacy of antimicrobial treatments against microbial cells and to elucidate their mechanism of action, it is interesting to conduct MP-FCM which provides a rapid acquisition of information related to the physiological state of cells and to interpret precisely their survival modes. Nonetheless it has to be kept in mind, that the cellular vital properties are not measured directly, but through the distribution of fluorescent probes or the conversion of substrates. Therefore, the interpretation of data could be different between Gram negative and Gram positive bacteria as well as between bacteria and yeast. Controls must be performed with caution and conclusions should always be drawn carefully.

Additional methods such as microscopy, spectroscopy, membrane modeling, or molecular biology techniques could complete and corroborate MP-FCM results. Viability and culturability potential of microbial cells could be determined by MP-FCM and plate counts, respectively. Depending on the antimicrobial treatment, its intensity, the strains, and the target cell physiological state, viability and culturability can correlate or not. If there was no correlation, this revealed the presence of a compromised VBNC subpopulation which appeared as viable with MP-FCM results but unable to grow on agar plates. The characterization by MP-FCM of such injured bacteria in food or in clinical applications might be critical in terms of their potential capacity to excrete toxic or food spoiling metabolites, to transfer genes (Ananta et al., 2004; Schenk et al., 2011; Ayari et al., 2013; Hong et al., 2015). In food associated microbial communities, gene transfer can have direct implications for human health via the acquisition of new metabolic traits: substrate utilization, bacteriocin, exopolysaccharide and biogenic amine production, immunity to bacteriophages and antibiotic resistance (Kelly et al., 2009; Rossi et al., 2014). This phenomenon could contribute to the distribution of acquired traits to intestinal bacteria (Rossi et al., 2014).

This review of MP-FCM methodologies to assess antimicrobial mechanism gives access to a set of protocols and antimicrobial mechanisms of actions following different treatments descriptions. This could allow the development of such methodology and thus give further information about impact of antimicrobial compounds or physical treatments on microbial cells.

## Author contributions

All authors listed, have made substantial, direct and intellectual contribution to the work and approved it for publication.

### Conflict of interest statement

The authors declare that the research was conducted in the absence of any commercial or financial relationships that could be construed as a potential conflict of interest.
